# Dilemmas about instructions for administering drugs and indications for their use: is there negative effect of pharmaceutical industry?

**DOI:** 10.1186/s40169-020-0267-0

**Published:** 2020-01-30

**Authors:** Enver Zerem

**Affiliations:** 1 Department of Medical Sciences, The Academy of Sciences and Arts of Bosnia and Herzegovina, Bistrik 7, 71000 Sarajevo, Bosnia and Herzegovina; 2 Department of Internal Medicine, Cantonal Hospital Safet Mujić, 88000 Mostar, Bosnia and Herzegovina

**Keywords:** Instructions for administering drugs, Impact of pharmaceutical industry, Statins, Anticoagulants, Insulin analogues

## Abstract

Instructions for administering some drugs and indications for their use raise certain dilemmas and controversies questioning the appropriateness of the treatment in this way. In this article, some controversies regarding the prescribing of statins in patients whose blood cholesterol level is normal and the use of anticoagulants in the elderly patients without blood clots prior to the treatment are described. Also, it is discussed about some controversies regarding the use of the insulin analogues in the treatment of patients with diabetes mellitus.

Over the past decades, the pharmaceutical industry has achieved great successes resulting in the emergence of new drugs that contributing to improvements in the treatment of a large number of diseases. However, instructions for administering some drugs and indications for their use raise certain dilemmas and controversies questioning the appropriateness of the treatment.

For example, statins are drugs that lower blood cholesterol level. However, current opinion is that they protect the blood vessel walls by inhibiting inflammation. Therefore, they are given to anyone at risk of cardiovascular diseases regardless of whether their blood cholesterol level is elevated or not. The rationale for using statins, in patients with normal blood cholesterol level, is highly debatable because it is very difficult to imagine a study design that could objectively confirm this. Also, it is very difficult or impossible to assess the effect of statin administration in patients with normal blood cholesterol level prior to the treatment (is the expected outcome of statin administration to reach blood cholesterol level equal to zero!?). In addition, some studies suggest that statins can increase glycaemic levels and lead to type 2 diabetes [1].

It is similar situation with the increasing use of anticoagulant drugs which are given to patients at high risk of blood clots, with intention to reduce the chances of developing serious conditions such as stroke and heart attack. Although, the use of anticoagulants may be advantageous for the cardiovascular or the cerebrovascular system, long-term or lifelong administration of anticoagulant drugs has undoubtedly a large number of negative effects on other organs with numerous consequential complications. It is known that anticoagulants do not dissolve the previously created blood clots but only prevent the formation of new ones. Therefore, the use of anticoagulants is controversial in the elderly without clots, because it is debatable whether it is more likely that anticoagulants could cause complications or that the clots would be formed if anticoagulants are not taken. At the very least, the approach to this problem should be more rigorously individualized [2, 3]. Particularly questionable is the use of new anticoagulants (rivaroxaban, dabigatran, apixaban and edoxaban) since the risks associated with those, in routine use, cannot be monitored, and could only be reacted upon once the complications arise.

Particularly controversial is the use of new forms of insulin, the so-called insulin analogues, as a new therapy in the treatment of patients with Diabetes Mellitus. The discovery of insulin is one of the most significant medical discoveries of the twentieth century. A fourteen-year-old boy, Leonard Thompson, is the first person to be injected with an isolated bovine insulin extract on January 11, 1921. Since then, insulin has saved millions of lives and fundamentally altered the course and prognosis of diabetes. Animal insulins (bovine and porcine) remained for a long time as the only insulins. The first genetically synthetic human insulin was produced in 1977, and its commercial sale began in 1988 (Eli Lilly). During the 1990s, new research led to the discovery of a new generation of insulin, the so-called human insulin analogues, which are claimed to have better characteristics than human insulins; faster or longer action, less hypoglycaemias and so on.

It is clear that the inclusion of porcine insulin instead of bovine insulin in the therapeutic protocol of Diabetes Mellitus was logical (because it is more similar to human) as well as that the inclusion of human insulin is more appropriate than that of porcine insulin, but the benefits of insulin analogues (faster and longer acting) in comparison to human insulin are debatable. For, it is not logical that insulin analogous are more similar to natural human insulins, nor that the aforementioned benefits (faster and longer acting) can justify the great difference in price. It is questionable why it is important to have insulin that acts faster and longer than human (than insulin produced by the normal human pancreas). Theoretically speaking, if it would be true that insulin analogues have a significantly better effect compered to human insulin, we would face an absurd situation whereby patients treated with insulin analogues have more optimal glycaemic control than healthy individuals.

It is a well-known that new insulin formulations most often appear when the previous formulations of the same manufacturer are about to lose patent protection rights and when it is logical to expect a significant fall in the price of the drug. The most striking example, in this regard, is the inclusion of Glargine 300 IU as a new drug instead of Glargine 100 IU. Hence, it is logical to conclude, that the improvement of the treatment of patients was not in the foreground, but an attempt of the price protection of their drug (Glargine 100 IJ) since its patent rights have expired and the emergence of new generic parallels would inevitably reduce its price. By establishing a very similar, virtually the same “new” drug (Glargine 300 IJ) as a substitute for the “old” one (Glargine 100 IJ) the patent rights are continued which prevents the impact of the new generic parallels on lowering the drug price. It goes a step further here, since, in most countries, the new drug is registered as few percent cheaper than the old one, but the drug packaging is reduced by about 11% (1500 IJ versus 1350 IJ), practically meaning that the price has increased.

A particular problem is the association of insulin analogues with malignancies. Some studies have found that this correlation exists [4, 5]. Others no correlation in their study design (without a clear claim that it did not exist), with offering the most common explanation that it was a short patient follow-up time [6, 7].

## Conclusion

The pharmaceutical industry has achieved great successes resulting in the emergence of new drugs that contributing to improvements in the treatment of a large number of diseases. However, instructions for administering some drugs and indications for their use raise certain dilemmas and controversies questioning the appropriateness of the treatment in this way.

## Data Availability

Not applicable.
